# Changes in plasma hydrogen sulfide are associated with cognitive improvements in schizophrenia patients treated with atypical antipsychotics

**DOI:** 10.3389/fpsyt.2026.1789058

**Published:** 2026-03-30

**Authors:** You-Chang Huang, Yi-Heng Li, Long Li, Jian-Wen Xiong, Bo Wei, Yuan-Jian Yang, Ying Zhao, Hai-Yan Zhan

**Affiliations:** 1 Department of Psychiatry, The Third Hospital of Fuzhou City, Fuzhou, China; 2 Biological Psychiatry Laboratory, Jiangxi Mental Hospital & Affiliated Mental Hospital, Jiangxi Medical College, Nanchang University, Nanchang, China; 3 Department of Psychiatry, The Third People’s Hospital of Ji′an City, Ji′an, China; 4 Department of Pharmacy, Union Hospital, Tongji Medical College, Huazhong University of Science and Technology, Wuhan, China

**Keywords:** atypical antipsychotics, cognitive function, hydrogen sulfide (H2S), plasma, schizophrenia

## Abstract

**Background:**

Hydrogen sulfide (H₂S) acts as a neuromodulator in the brain and is shown to be associated with cognitive impairments in schizophrenia. Atypical antipsychotics can provide cognitive benefits for schizophrenia patients. This prospective observational study aims to investigate whether H_2_S signaling is involved in the cognitive improvement effects of atypical antipsychotics in patients with schizophrenia.

**Methods:**

A total of 25 schizophrenia patients with acute exacerbation who completed follow-up and 28 healthy controls were included in this study. Psychopathological symptoms and cognitive function were assessed using the Positive and Negative Syndrome Scale (PANSS) and a neuropsychological test battery, respectively. Plasma H_2_S levels were determined using high-performance liquid chromatography (HPLC).

**Results:**

We found that compared with normal controls, schizophrenia patients exhibited poorer cognitive function and lower plasma H_2_S levels at baseline (*p* < 0.05). After two months of atypical antipsychotic treatment, the patients showed significant improvements in processing speed, working memory, visuospatial memory, attention, and executive function (all *p* < 0.01). At the same time, plasma H_2_S levels in patients after treatment were significantly elevated compared to baseline (0.918 ± 0.036 vs. 0.712 ± 0.023 µmol/L; *t* = 6.807, *p* < 0.001). Correlation analysis revealed that the increase in H_2_S was significantly associated with improvements in working memory (r = 0.291, *p* = 0.005) and visuospatial memory (r = 0.227, *p* = 0.016).

**Conclusion:**

Our findings demonstrated that cognitive improvement in patients with schizophrenia after treatment with atypical antipsychotics is correlated with an increase of plasma H₂S levels, suggesting that H_2_S signaling is involved in the pathophysiological process of cognitive impairment in schizophrenia.

## Introduction

Schizophrenia is a common severe mental disorder with an aetiological and therapeutic challenge (1). Patients with schizophrenia exhibit perceptual, thinking, and behavioral dysfunctions, usually accompanied by varying degrees of cognitive deficits, including impairments in processing speed, attention, visual memory, verbal learning, working memory, and executive function (2). Cognitive impairment represented a core feature of schizophrenia and has profound negative effects on social functioning and quality of life in individuals with this disorder (3). Therefore, treating cognitive impairment is crucial for schizophrenia patients to achieve good clinical outcomes.

Antipsychotic medications are the main treatment strategy for schizophrenia. Antipsychotic medications are generally classified into two main categories: first-generation agents (FGAs), such as chlorpromazine, perphenazine and haloperidol, and second-generation agents (SGAs), such as clozapine, risperidone, and olanzapine. FGAs primarily alleviate positive symptoms, such as delusions and hallucinations (4), while SGAs, also referred to as atypical antipsychotics, exert significant effects on positive, negative and cognitive symptoms in schizophrenia (5). Previous studies have shown that SGAs treatment can produce beneficial effects of small to moderate effect size on cognition including executive functions, verbal fluency, working memory, while long-term use of FGAs would lead to a significant decline in executive function, verbal learning and memory (6, 7). Thus, SGAs have an advantage over FGAs regarding cognitive function during a medium-term treatment for schizophrenia.

Hydrogen sulfide (H_2_S), which was known to be a toxic gas, has been recognized as the third gaseous signaling molecule following nitric oxide (NO) and carbon monoxide (CO) (8). As an important neuromodulator, H_2_S plays a key role in regulating various physiological processes in both the brain and peripheral systems (9, 10). Brain H_2_S is mainly produced by cystathionine-β-synthase (CBS) and 3-mercaptopyruvate sulfurtransferase (3-MST) (11). It modulates synaptic transmission and plasticity which is essential for cognition, and exhibits anti-inflammatory effects which are helpful in the progress of the neurodegenerative condition (12, 13). Dysfunction in H_2_S signaling has been shown to contribute to cognitive impairment associated with various neurological disorders, including Alzheimer’s disease (AD), Parkinson’s disease (PD), and cerebral ischemia (14).

Recent studies have demonstrated that alterations of H_2_S signaling are implicated in the pathophysiology of cognitive impairments in schizophrenia (15–17). Previous study has reported that plasma H_2_S levels were significantly decreased in patients with schizophrenia and correlated with the cognitive impairment symptoms of the patients (16). Du et al. showed H_2_S content was significantly decreased in the plasma of schizophrenia patients and in the plasma and hippocampal tissue of schizophrenia model rats, while supplementation of H_2_S in model rats can alleviate cognitive and behavioral abnormality by inducing S-sulfhydration of apoptotic proteins (15). Furthermore, it was shown that serum native thiol and the total thiol concentration were significantly decreased in schizophrenia patients, and the disulfide/native thiol ratio in patients who have been using medication was significantly higher than controls who have not been using medication (18). However, it is currently unknown whether atypical antipsychotic treatment would affect plasma H_2_S levels in patients with schizophrenia, and if it does, whether changes in plasma H_2_S are related to cognitive improvement of patients. In this study, we conducted a prospective observation trial to investigate the effects of atypical antipsychotics on plasma H_2_S and its relationship with cognitive improvements in patients with schizophrenia.

## Materials and methods

### Subjects

This open-label, prospective observational study included 25 schizophrenia patients with acute exacerbation from Jiangxi Provincial Psychiatric Hospital (13 males and 12 females). The diagnosis of schizophrenia was established by two psychiatrists according to the Diagnostic and Statistical Manual of Mental Disorders, Fourth Edition (DSM-IV). Participants were aged between 18 and 50 years, with baseline Positive and Negative Syndrome Scale (PANSS) total scores ranging from 60 to 120. Exclusion criteria included any additional axis I or axis II DSM-IV diagnosis, pregnancy or the presence of other severe physical diseases including cardiac or cerebral infarction within the past 3 months. All patients were either antipsychotic-naive or had not received any antipsychotic medication for at least 3 months prior to enrollment. 27 healthy controls (12 males and 15 females) were recruited from the local community, matched to the patient group based on age, sex, years of education, and body mass index (BMI). Individuals with a personal or family history of mental disorders were excluded from the control group.

The study procedures complied with the ethical standards of the Declaration of Helsinki. Ethical approval was granted by the Institutional Review Board at Jiangxi Mental Hospital. Prior to any study procedures, voluntary written informed consent was secured from every participant or, when applicable, their legal guardians.

### Medication and evaluations of clinical symptom and cognitive function

The type of atypical antipsychotic was chosen based on the patient’s demographic characteristics and clinical symptom features. The atypical antipsychotics included in this study were aripiprazole (n = 5), olanzapine (n = 4), risperidone (n = 9), and clozapine (n = 7). Patients receiving clozapine treatment met the criteria for treatment-resistant schizophrenia. These enrolled patients were treated with only one atypical antipsychotic during the study. Benzhexol was used only when patients exhibited significant extrapyramidal reactions. In clinical practice, the most commonly used treatment duration for patients with acute schizophrenia is 8 weeks. In this study, only patients who completed the 8-week follow-up were included.

The severity of the patient’s psychiatric symptoms was assessed at baseline and follow-up using the Positive and Negative Syndrome Scale (PANSS). The assessments were conducted by two psychiatrists who had received training for consistent use of the PANSS before the study began. The interobserver correlation coefficient for the total PANSS score was greater than 0.80.

Participants’ cognitive functions were assessed using a comprehensive battery of neurocognitive tests that are well-established and validated for use in China (16). This assessment battery includes seven tests:

The trail making test part A (TMT-A): In this test, the participant was provided with a pencil and a worksheet containing randomly placed, numbered circles. The task required the participant to draw a single continuous line to connect the circles in sequential order, completing it as quickly and accurately as possible. The primary outcome measure is the total time taken to complete the task.

Brief Assessment of Cognition in Schizophrenia (BACS) Symbol Coding: In this test, participants were given a key containing 133 digit-symbol pairs and were required to transcribe the symbol corresponding to each digit as quickly and accurately as possible within a 120-second time limit. Scoring is based on the total number of symbols correctly transcribed within the allotted time.

Wechsler Memory Scale, Third Edition Spatial Span Test (WMS-III Spatial Span): In this test, participants are presented with a board containing ten irregularly arranged blocks. The task requires participants to observe and then recall the sequence in which the administrator points to the blocks, reproducing it both in forward and backward order. Each sequence level allows for two attempts. The final score corresponds to the participant’s performance on these recall trials.

Brief Visuospatial Memory Test-Revised (BVMT-R): In this test, participants are required to reproduce six geometric figures from memory. Each figure is shown for 10 seconds in sequence, and this process is repeated for three consecutive trials. Participants are asked to draw the figures as accurately as possible in the designated area of the answer booklet. Scoring is based on the total number of correctly reproduced figures.

Hopkins Verbal Learning Test-Revised (HVLT-R): This is a verbal memory test in which participants are shown a list of 12 words consisting of three different semantic categories. The word list is presented three times, followed by a 25 to 30-minute delay. Afterwards, participants are instructed to recall as many words as possible. Test scores are determined by the total number of words correctly recalled.

Continuous Performance Test-Identical Pairs (CPT-IP): In this test, a sequence of numbers (lengths of 2, 3, and 4) flashes on the screen. Participants are only supposed to click the mouse when the same sequence appears twice in a row. Out of a total of 270 random presentations, targets (sequences that need to be clicked) appear approximately 90 times. Responses to non-target sequences are considered false alarms.

Stroop Color-Word Test (SCWT): This Stroop test includes three conditions: the word condition (reading color words in black ink), the color condition (naming the ink color of ‘X’s), and the critical interference condition (naming the ink color of color words printed in non-matching colors). In each 45-second trial, participants are asked to verbally process 100 items as quickly as possible, with the dependent variable being the number of correct responses under each condition.

### Plasma H_2_S measurement

Blood samples were collected from overnight-fasted patients. Whole blood was drawn into EDTA-containing tubes and centrifuged at 3000 rpm for 5 min at 4 °C. The resulting plasma was aliquoted and stored at −80 °C until analysis.

**Figure 1:**
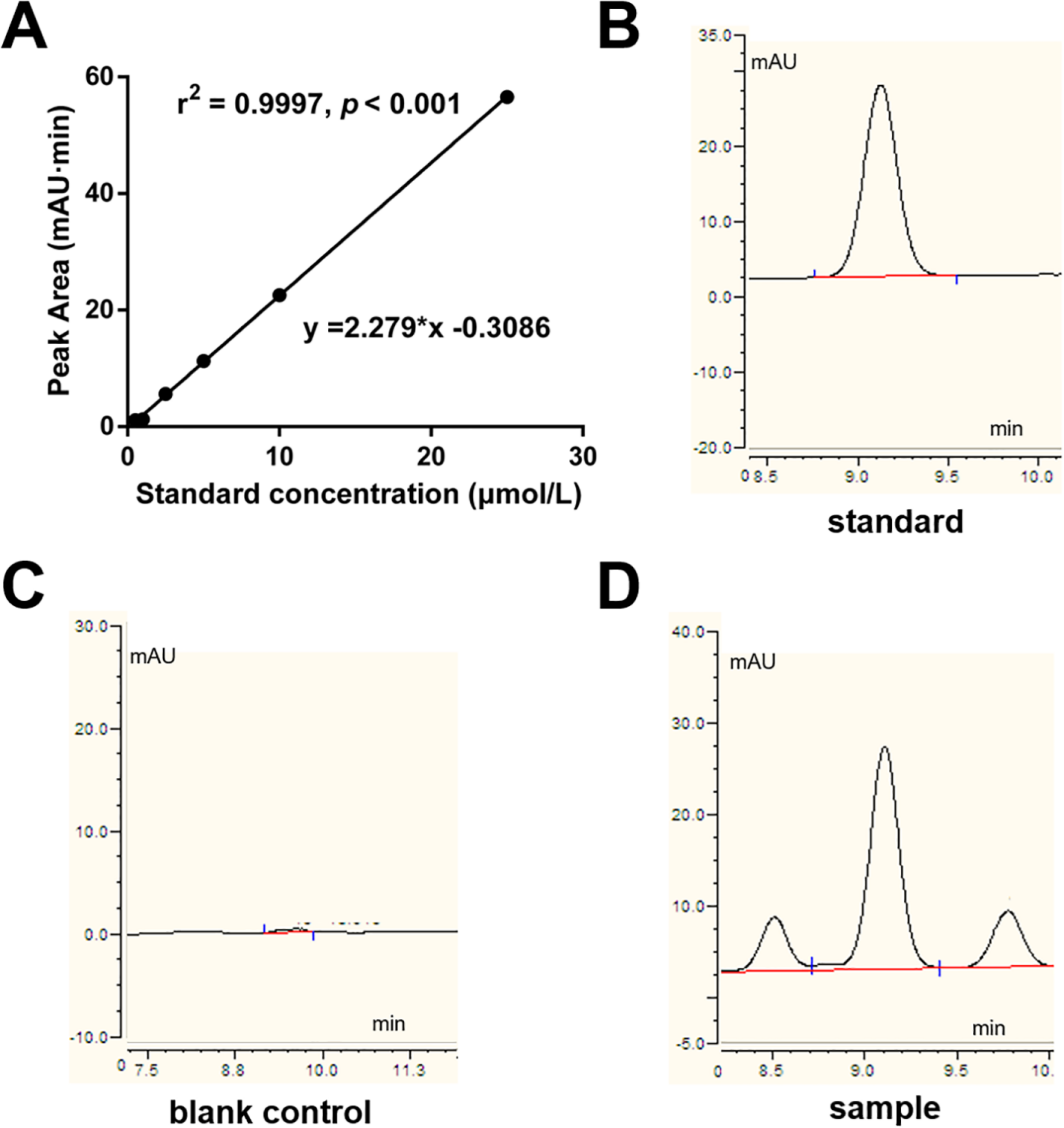
HPLC analysis of H_2_S. **(A)** Calibration curve for H_2_S quantification. **(B–D)** Representative chromatograms of H_2_S standard **(B)**, blank control **(C)**, and a plasma sample **(D)**.

Plasma H_2_S levels were quantified using a derivatization method based on monobromobimane (MBB) combined with reverse-phase high-performance liquid chromatography (RP-HPLC), as previously described (16). In brief, free H_2_S in plasma reacts with an excess of MBB to form sulfide-bimane, a stable fluorescent product. Each 30 μL plasma sample was mixed with 70 μL of 100 mM Tris-HCl buffer (pH 9.5, containing 0.1 mM DTPA), followed by the addition of 50 μL of 10 mM MBB. After 30 min, the reaction was terminated with 50 μL of 200 mM 5-sulfosalicylic acid. Following centrifugation, the supernatant was injected into an Agilent 1220 HPLC system equipped with an Agilent ZORBAX Eclipse XDB-C18 column. The mobile phase consisted of solvent A (0.1% trifluoroacetic acid in water) and solvent B (0.1% trifluoroacetic acid in acetonitrile) with a gradient elution program at a flow rate of 0.75 mL/min. Quantification was performed using a calibration curve constructed from sulfide-bimane standards. The calibration curve was constructed by plotting peak area against H_2_S concentration, yielding a typical regression equation of y = 2.279x − 0.3086 with a correlation coefficient (R^2^) of 0.9997, indicating excellent linearity over the concentration range tested (**Figure 1A**). Representative chromatograms of standard, blank control and sample are shown in **Figures 1B–D**.

### Statistical analysis

SPSS 22.0 and GraphPad Prism 8.0 software were used to perform statistical analysis and draw for all data separately. Student’s *t*-test was used to compare two groups of quantitative variables that followed a normal distribution, while the Mann-Whitney U test was applied to variables that do not follow a normal distribution. The Shapiro-Wilk normality test was used to assess the distribution of quantitative data. For qualitative variables, Fisher’s exact test was utilized, especially when the sample size was small. Comparisons of plasma H_2_S levels and scale scores at two time points in the same patients—baseline and after 8 weeks of atypical antipsychotic treatment—were performed using the paired *t*-test (for normally distributed data) or the Wilcoxon matched-pairs signed rank test (for non-normally distributed data). One-way analysis of variance (ANOVA) was applied to compare H_2_S concentration before and after treatment across different types of atypical antipsychotics. Spearman correlation analysis was conducted to examine the relationship between changes in cognitive test scores and H_2_S levels. All statistical tests were two-sided, and a *p*-value of less than 0.05 was considered statistically significant.

## Results

### Demographic and clinical characteristics and cognitive performance of patients and controls

A total of 25 schizophrenia patients with acute exacerbation who were received monotherapy with an atypical antipsychotic was included in this study. These patients completed the baseline assessment as well as an 8-week follow-up. The selection of the specific atypical antipsychotic was individualized based on the patient’s clinical characteristics, including age, gender, and symptom presentation. Baseline demographic and clinical characteristics for both patients and normal controls are presented in **Table 1**. The patient and control group were well-matched in age, gender, years of education, and BMI (all *p* > 0.05). Cognitive assessments demonstrated significantly poorer performances across all tested domains in schizophrenia patients compared to controls (all *p* < 0.05). The levels of plasma H_2_S were measured in both groups and the results show that plasma H_2_S levels were significantly decreased in schizophrenia patients compared to normal controls (0.712 ± 0.023 vs. 0.923 ± 0.026 μmol/L; t = 6.602, *p* < 0.001).

**Table 1:** Demographic and clinical characteristics of the patient and control group at baseline.

Variables	Patients	Controls	t/U	*p*
Age (years)	30.88 ± 1.693	33.11 ± 1.969	1.617	0.112
Gender (M/F)	13/12	12/15	0.297	0.586
Education (years)	10.20 ± 1.010	11.00 ± 0.900	0.593	0.556
BMI (kg/m2)	21.62 ± 0.4172	20.97 ± 0.390	1.139	0.260
Duration of illness (years)	6.464 ± 1.068	NA		
PANSS				
Total scores	80 [75-84]	NA		
Positive subscore	22 [20-26]	NA		
Negative subscore	12 [11-14]	NA		
General psychopathology	44 [40-46]	NA		
Cognitive tests				
TMT-A	70 [48-85.5]	38 [32-45]	66.50	< 0.001
BASC-SC	31 [22-46.5]	66 [62-71]	19.50	< 0.001
WMS-III-SS	15.06 ± 0.542	16.76 ± 0.414	2.507	0.016
HVLT-R	19.48 ± 1.153	26.74 ± 0.924	4.948	< 0.001
BVMT-R	20 [19-24]	28 [13-38]	196.5	0.009
CPT-IP	1.177 [0.803-2.062]	3.459 [2.351-4.238]	59.00	< 0.001
Stroop word score	53.16 ± 3.100	87.11 ± 1.591	9.958	< 0.001
Stroop color score	35.20 ± 2.787	51.11 ± 1.752	4.910	< 0.001
Stroop color-word score	14 [11.5-27.5]	38 [33-40]	81.50	< 0.001
**Plasma H_2_S (µmol/L)**	0.712 ± 0.023	0.923 ± 0.026	6.602	< 0.001

BMI, body mass index; PANSS, Positive and Negative Syndrome Scale; TMT-A, trail making task part A; BACS-SC, brief assessment of cognition in schizophrenia-symbol coding; WMS-III-SS, Wechsler memoryscale-3rdedition-spatial span; HVLT-R, Hopkins verbal learning test-revised; BVMT-R, brief visual-spatial memory test-revised; CPT-IP, continuous performance test-identical pairs. NA, not applicable.

### Clinical and neurocognitive outcomes of atypical antipsychotic in patients

The primary endpoint was the change in PANSS scores and cognitive test results from baseline to week 8. **Table 2** presents the clinical efficacy and neurocognitive effects of atypical antipsychotics in patients with schizophrenia. After the study was completed, significant improvements were observed in patients in the total PANSS score as well as in each subscale score (paired *t*-test; all *p* < 0.001). Furthermore, patients who received 8-week treatment with atypical antipsychotic showed significantly improved scores on the TAM-A, BACS-SC, WMS-III, BVMT-R, CPT-IP, and Stroop Color-Word Test (all *p* < 0.05), except for HVLT-R.

**Table 2:** Comparison of PANSS and cognitive scores between baseline and endpoint in patients.

Variables	Baseline	Endpoint	t/U	*p*
PANSS				
Total scores	80 [75-84]	34 33-36.5]	-325.0	< 0.001
Positive subscore	22 [20-26]	7 [7-7]	-325.0	< 0.001
Negative subscore	12 [11-14]	7 [7-8]	-300.0	< 0.001
General psychopathology	44 [40-46]	19 [18-20.5]	-325.0	< 0.001
COGNITIVE FUNCTION				
TMT-A	70 [48-85.5]	50 [42-69]	-300.0	< 0.001
BACS-SC	31 [22-46.5]	36 [29-49.5]	2.373	0.026
WMS-III-SS	15.06 ± 0.542	18.35 ± 0.689	12.01	< 0.001
HVLT-R	20 [14.5-24]	21 [15.5-25]	1.077	0.081
BVMT-R	21.24 ± 0.926	26.72 ± 0.919	10.67	< 0.001
CPT-IP	1.385 ± 0.192	1.774 ± 0.113	2.507	0.019
Stroop word score	53.16 ± 3.100	68.44 ± 2.831	7.505	< 0.001
Stroop color score	35.20 ± 2.787	41.68 ± 2.374	3.255	0.003
Stroop color-word score	14 [11.5-27.5]	23 [15.5-31.5]	325	< 0.001
**Plasma H_2_S (µmol/L)**	0.712 ± 0.023	0.918 ± 0.036	6.807	< 0.001

### Effects of different atypical antipsychotic on plasma H_2_S levels

**Figure 2:**
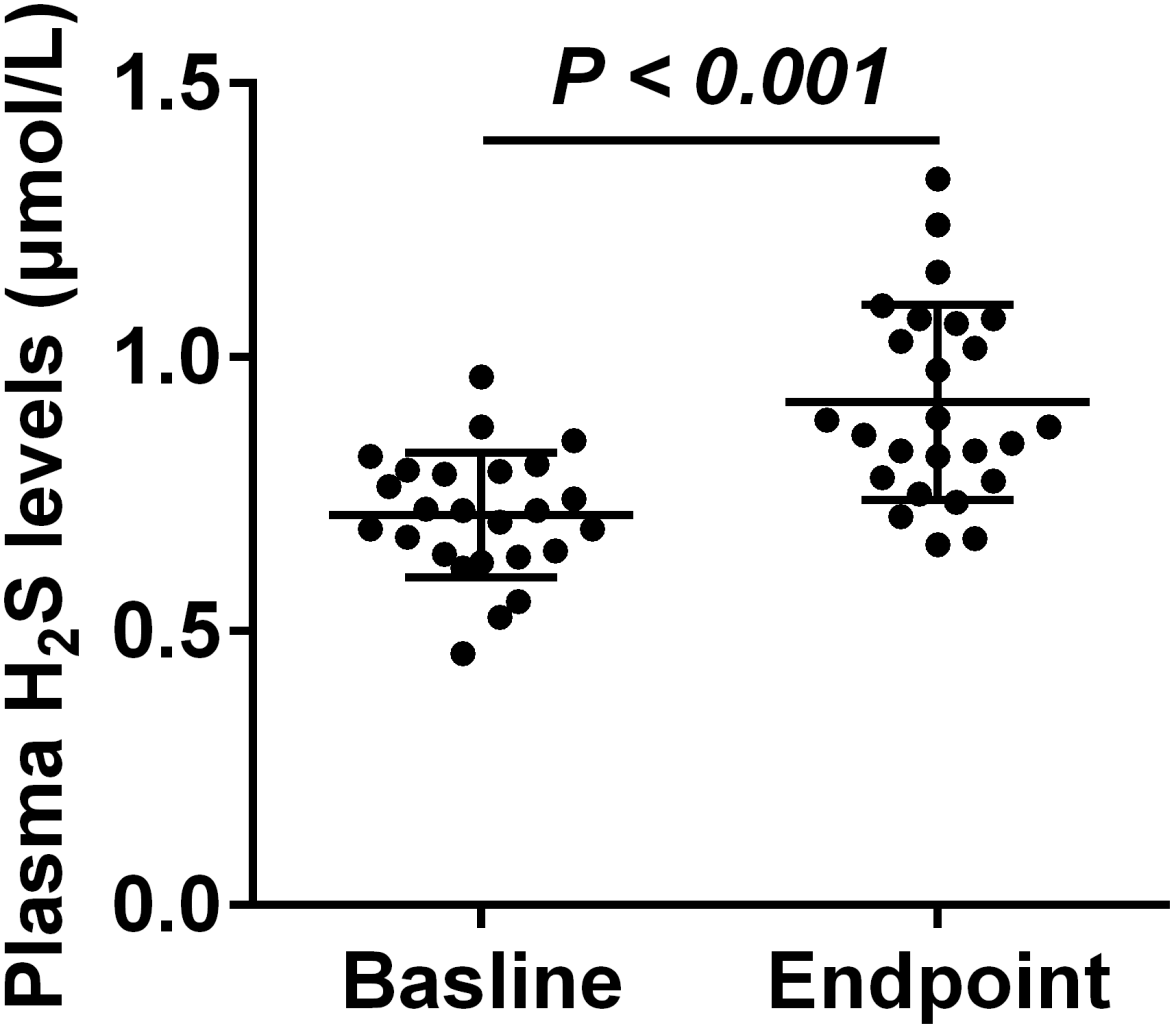
Comparison of plasma H_2_S levels in schizophrenia patients between baseline and the endpoint. Scatter plot showing the plasma H_2_S levels at both time points. Data are presented as a scatter plot, with the black bars representing the sample means.

Following 8 weeks of atypical antipsychotic therapy, a significant increase in plasma H_2_S levels from baseline was observed in patients (0.712 ± 0.023 vs. 0.918 ± 0.036 μmol/L; t = 6.807, *p* < 0.001) (**Figure 2**). The levels of plasma H_2_S in schizophrenia patients treated with atypical antipsychotic were comparable to those of control group (*p* > 0.05). As patients received different antipsychotic (aripiprazole [n = 5], olanzapine [n = 4], risperidone [n = 9], and clozapine [n = 7]), we conducted statistical comparisons to determine if the change of H_2_S level varied significantly across these four treatment groups. We found that all medication regimens significantly increased plasma H_2_S levels in patients (all *p* < 0.05), and no significant differences were detected among the treatment groups either before the treatment (F_(3, 21)_ = 1.023, *p* = 0.403) or after the intervention (F_(3, 21)_ = 0.660, *p* = 0.586) (**Table 3**).

**Table 3:** Plasma H_2_S levels before and after treatment with different atypical antipsychotic.

Variables	Baseline H_2_S (μmol/L)	Endpoint H_2_S (μmol/L)	*t*	*p*
Aripiprazole (n = 5)	0.724 ± 0.029	0.913 ± 0.060	3.824	0.019
Olanzapine (n = 4)	0.794 ± 0.071	1.009 ± 0.042	3.949	0.029
Risperidone (n = 9)	0.699 ± 0.032	0.932 ± 0.067	4.098	0.003
Clozapine (n = 7)	0.674 ± 0.053	0.851 ± 0.083	2.467	0.049
F	1.023	0.660		
*p*	0.403	0.586		

### Association between cognitive improvement and the alteration in plasma H_2_S levels

**Figure 3:**
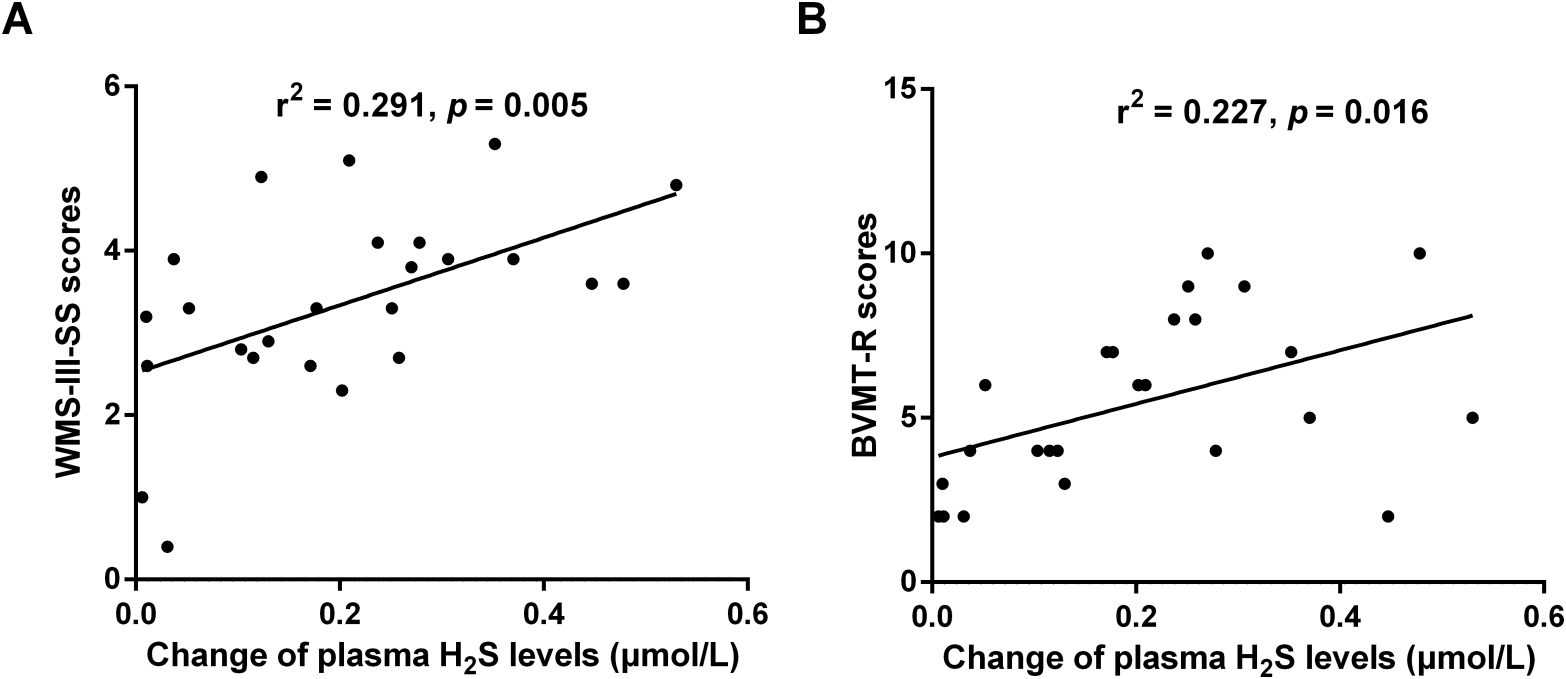
The correlation between the change of plasma H_2_S levels and the improvement of WMS-III-SS scores **(A)** or BVMT-R scores **(B)** in patients with atypical antipsychotic treatment.

Spearman correlation analysis was conducted to assess whether changes in plasma H_2_S level were associated with clinical improvements. The results show that the increases in plasma H_2_S were not significantly correlated with the changes in PANSS total score (r = 0.038, *p* = 0.347), positive subscale (r = 0.012, *p* = 0.603), negative subscale (r = 0.004, *p* = 0.756), or general psychopathology score (r = 0.129, *p* = 0.078) in patients before and after treatment. Subsequently, we further examined the relationship between change in plasma H_2_S level and the cognitive improvement. As shown in **Figure 3**, a significant positive correlation was observed between the increase in plasma H_2_S level and the improvement in WMS-III-SS (r = 0.291, *p* = 0.005) and BVMT-R score (r = 0.227, *p* = 0.016). No significant correlations were found for the remaining cognitive measures (TMT-A: *p* = 0.708; DSCT: *p* = 0.6569; CPT: *p* = 0.318; HVLT-R: *p* = 0.586; Stroop word score: *p* = 0.067; Stroop color score: *p* = 0.105; Stroop color-word score: *p* = 0.180). After controlling for change in PANSS total score, the correlation between H_2_S change and improvement in WMS-III-SS (r = 0.281, *p* = 0.006) and BVMT-R score (r = 0.212, *p* = 0.021) remained significant. These results suggest that the increase in plasma H_2_S was associated with enhanced working memory and visuospatial memory in patients receiving atypical antipsychotic treatment.

## Discussion

This study aimed to investigate the effects of atypical antipsychotics on psychotic symptoms, cognitive function and plasma H_2_S levels in schizophrenia patients. We found that patients with schizophrenia exhibited significantly lower plasma H_2_S levels compared to healthy controls. After 8 weeks of treatment, significant improvements were observed in both psychotic symptoms and cognitive performance, accompanied by an increase of plasma H_2_S levels, and the elevation of plasma H_2_S levels was significantly correlated with the cognitive improvement in patients. These findings suggested that abnormal H_2_S signaling may contribute to the cognitive impairments in patients with schizophrenia.

Cognitive impairment was a well-established core symptom of schizophrenia (19). In numerous cognitive tests, patients with schizophrenia score below the normal level on average (20, 21). Consistent with previous reports (7, 16), our present study utilized a series of cognitive tests and demonstrated significant cognitive impairments in schizophrenia patients across multiple domains including processing speed, working memory, attention, and executive function. After two months of atypical antipsychotic treatment, the cognitive function of patients showed varying degrees of improvement. This observation is consistent with previous studies and confirms that atypical antipsychotics could improve cognitive function in patients with schizophrenia (22, 23).

H_2_S is naturally produced in mammalian tissues and exerted a range of biological and physiological effects (24, 25). Notably, accumulating evidence support its role in neuromodulation (26, 27). H_2_S has been reported to facilitate the induction of hippocampal long-term potentiation (28, 29), upregulate the expression of hippocampal GABA-B receptors (30, 31), and modulate calcium and pH homeostasis in neuronal and glial cells (32–35). Furthermore, H_2_S exhibited neuroprotective properties by shielding neurons from oxidative stress, indicating therapeutic potential for neurodegenerative disorders such as Alzheimer’s and Parkinson’s diseases (36). It also demonstrated antidepressant and anxiolytic effects in models of depression and anxiety (37). Consistent with previous studies (15, 16), our current study found that plasma H_2_S levels were significantly reduced in patients with schizophrenia. Additionally, it was shown that supplement with H_2_S can mitigate schizophrenia-like behaviors in rodents by modulating apoptosis via S-sulfhydration (15). These data suggest that abnormal H_2_S signaling may contribute to the pathogenesis of schizophrenia and has the potential to be a therapeutic target for schizophrenia.

In recent years, there has been growing interest in identifying biomarkers associated with schizophrenia, particularly for quantifiable aspects such as cognitive function. Cytokines, including interleukin-1 and -6 (IL-1, IL-6) as well as tumor necrosis factor (TNF), have been found to be associated with cognitive decline and other negative symptoms (38). Elevated prolactin levels in patients with early psychosis were associated with impaired processing speed, independent of antipsychotic medication use (39). Clinical evidence suggests that H_2_S might serve as a biomarker for Alzheimer’s disease and related dementias (40). Patients with Alzheimer’s disease, vascular dementia, or cerebrovascular diseases showed a significant decrease in plasma H_2_S level, and this decrease was correlated with the progression of cognitive decline (41). Experiments in rodents have shown that administration of H_2_S can improve cognitive function in various models of cognitive impairment, including cognitive deficits caused by homocysteine (42), lipopolysaccharide (43), traumatic brain injury (44), and diabetes (45). Our current study found that reduced plasma H_2_S level was significantly increased in schizophrenia patients after two months of treatment with atypical antipsychotic. Moreover, the recovery of H_2_S levels was associated with the cognitive improvement (working memory and visual learning memory) in patients, suggesting that plasma H_2_S may have potential in assessing cognitive function in schizophrenia patients.

Despite the suggestive results, some limitations of the current study should be noted. First, this is a prospective observational study, which may have selection bias. Caution should be taken when generalizing this conclusion to other populations. Second, we only measured H_2_S levels in plasma and did not measure them in cerebrospinal fluid (CSF). Although H_2_S can quickly cross cell membranes and the blood-brain barrier, it is still uncertain whether brain H_2_S levels change parallelly with the alteration of plasma H_2_S. Third, the sample size of this study is relatively small. Large-scale clinical studies are needed to replicate and validate this conclusion. Fourth, correlation analysis shows a relationship between cognitive improvement and increase of plasma H_2_S in patients with schizophrenia. However, the causal relationship between these two variables cannot be determined. Further studies in animals are needed to demonstrate this relationship. Finally, NO and CO are also gas signaling molecules closely related to cognitive function. Although previous studies have reported changes in NO in schizophrenia and its effects on cognitive function, simultaneously measuring these three gaseous signaling molecules—NO, CO and H_2_S—and analyzing their interactive effects on cognitive function in schizophrenia will help to more comprehensively understand the role of gaseous signaling molecules in schizophrenia.

In conclusion, our study conducted a prospective, open-label, 8-week observational trial to investigate the effects of atypical antipsychotics on cognitive function and plasma H_2_S levels in patients with schizophrenia. We show that atypical antipsychotic medication significantly improved cognitive function and elevated plasma H_2_S levels in schizophrenia patients with acute exacerbation. The increase in H_2_S level was positively correlated with cognitive improvement in patients, suggesting that H_2_S signaling might be involved in the pathophysiological process of cognitive impairment in schizophrenia.

## Data Availability

The raw data supporting the conclusions of this article will be made available by the authors, without undue reservation.
